# Structural and Elemental Analysis of the Freshwater, Low-Mg Calcite Coralline Alga *Pneophyllum cetinaensis*

**DOI:** 10.3390/plants9091089

**Published:** 2020-08-24

**Authors:** Federica Ragazzola, Regina Kolzenburg, Jurgita Zekonyte, Sebastian Teichert, Chulin Jiang, Ante Žuljević, Annalisa Caragnano, Annalisa Falace

**Affiliations:** 1 Institute of Marine Science, University of Portsmouth, Portsmouth PO4 9LY, UK; regina.kolzenburg@port.ac.uk; 2 School of Engineering, University of Portsmouth, Portsmouth PO1 3DJ, UK; jurgita.zekonyte@port.ac.uk (J.Z.); chulin.jiang@port.ac.uk (C.J.); 3 GeoZentrum Nordbayern, Friedrich-Alexander-Universität Erlangen-Nürnberg, 91054 Erlangen, Germany; sebastian.teichert@fau.de; 4 Institute of Oceanography and Fisheries, 21000 Split, Croatia; zuljevic@izor.hr; 5 Department of Life Sciences, University of Trieste, 34127 Trieste, Italy; annalisacaragnano@hotmail.com (A.C.); falace@units.it (A.F.)

**Keywords:** low-Mg calcite, element composition, structural integrity, freshwater

## Abstract

Coralline algae are one of the most diversified groups of red algae and represent a major component of marine benthic habitats from the poles to the tropics. This group was believed to be exclusively marine until 2016, when the first freshwater coralline algae *Pneophyllum cetinaensis* was discovered in the Cetina River, southern Croatia. While several studies investigated the element compositions of marine coralline algal thalli, no information is yet available for the freshwater species. Using XRD, LA-ICP-MS and nano indentation, this study presents the first living low-Mg calcite coralline algae with Mg concentrations ten times lower than is common for the average marine species. Despite the lower Mg concentrations, hardness and elastic modulus (1.71 ± 1.58 GPa and 29.7 ± 18.0 GPa, respectively) are in the same range as other marine coralline algae, possibly due to other biogenic impurities. When compared to marine species, Ba/Ca values were unusually low, even though Ba concentrations are generally higher in rivers than in seawater. These low values might be linked to different physical and chemical characteristics of the Cetina River.

## 1. Introduction

Coralline algae are the third most diversified group of red algae [1] with a global distribution from the high latitudes to the tropics [2,3]. They provide important ecosystem services as dominant autotrophic calcifiers in arctic and subarctic regions [4,5], consolidators of coral reefs [6] and builders of rhodolith beds, coralligenous bioconstructions and intertidal “rims” [7,8]. They have also been acknowledged for their role as carbon sinks, due to their high uptake, assimilation and therefore storage potential [9]. 

Recently, coralline algae have received renewed attention in the context of global change, due to the suggested vulnerability of their high-Mg calcite skeleton [6]. The genus *Pneophyllum* encompasses 17 species that are widespread in marine and some in transitional environments [10]. In 2016, the first freshwater coralline alga *Pneophyllum cetinaensis* Kaleb, Žuljević & Peña was discovered [11]. *Pneophyllum cetinaensis* and the other *Pneophyllum* species from European Atlantic and Mediterranean coasts are distinguished on a morpho-anatomical basis by differences in development, dimension, and organization of the crusts. It is assumed that the opportunistic nature of the brackish-water ancestor of *Pneophyllum cetinaensis*, together with the specific chemo–physical characteristics of the Cetina River (karst system), allowed the biome transition of this taxa [11]. There are several bottlenecks for the successful transition of coralline algae across the marine–freshwater boundary caused by chemo–physical obstacles. Among these, the reduced salt concentration in freshwater is one of the major impediments, challenging the maintenance of osmotic homeostasis. Additionally, the relative lack of Ca^2+^ ions is critical to coralline algae due to their absorption of calcium from surrounding waters for calcification [12]. The process of calcification in coralline algae is dependent not only on photosynthetic activity but also on inorganic carbon concentrations and Mg/Ca ratios in the water [13,14]. Magnesium is a common element in calcite and it has become customary to divide marine calcites in high-Mg calcite and low-Mg calcite based on a threshold of 3–4 mol% Mg. Many organisms have high-Mg calcite skeletons with magnesium contents ranging from 4% to 45% [15,16]. All coralline algae are so far listed among this high-Mg calcite organisms, with magnesium contents ranging from 10.5 to 16.4 wt.% MgCO_3_, with a mean of 13.1 wt.% MgCO_3_ [17], depending on the prevailing temperature and seawater chemistry [13,18,19]. The substitution of Ca^2+^ by Mg^2+^ is critical since it affects the calcite lattice geometry and solubility. In biogenic high-Mg calcite, mechanical properties are enhanced compared to low-Mg calcite, conferring greater elastic modulus (E) and hardness (H) to the tissue with increasing Mg^2+^ concentrations [19]. The different concentrations of Mg^2+^ found in coralline algae, are not only driven by phylogeny [17], but also by changes in water temperature, based on the endothermic substitution of Mg^2+^ in calcite, favoring the Mg^2+^ substitution at higher temperatures [20]. Mg/Ca ratios have been shown to faithfully record temperature variations in a range of marine calcifiers [21,22,23], including coralline algae [24,25,26,27,28,29].

However, Mg^2+^ is not the only trace and minor element in biogenic carbonates considered to be a reliable proxy of past environments. Of particular interest are all the cations that can substitute Ca^2+^ in the crystal lattice such as Sr^2+^ and Ba^2+^ in aragonite (orthorhombic crystal structure) and Mg^2+^ and Ba^2+^ in calcite (trigonal–rhombohedral crystal structure [30]). The incorporation of these ions occurs, for some part [31,32], proportionally to the concentration of the dissolved element in the water and for some elements, such as Sr^2+^ and Mg^2+^, this incorporation is also thermodynamically controlled [33].

In marine settings, skeletal Ba/Ca ratios have proven to be a valuable proxy, providing information on coastal sediment transport, freshwater discharge, salinity, and nutrients distributions [34,35]. Barium concentrations are generally higher in rivers and lakes than in seawater as a result of chemical weathering in their catchments, and freshwater discharges have thus been recognized as important sources of barium in seawater [36]. 

This study presents the trace elemental composition and structural integrity of *Pneophyllum cetinaensis* and provides a very first insight in the skeletal characteristics of a freshwater coralline alga. Particular attention will be given to the elements that provide valuable proxies within marine coralline algae, being Ba/Ca for coastal sediment transport, freshwater discharge, salinity, and nutrient distributions and Sr/Ca, and Mg/Ca for temperature.

## 2. Results

### 2.1. XRD Analysis

**Figure 1:**
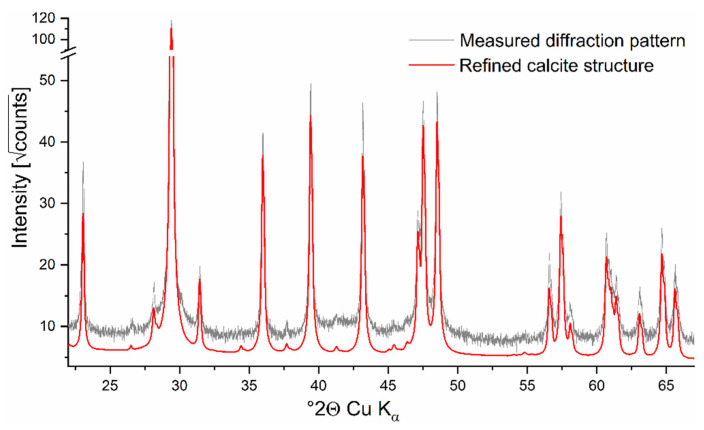
XRD spectra of *Pneophyllum cetinaensis* showing calcite as the only crystallite phase (*n* = 1).

Calcite was the only crystalline phase detected in the sample, as all XRD reflections were well covered by the calcite structure. Refinement of calcite resulted in a cry size (Lorentz contribution) of 94 nm and a microstrain (Gauss contribution) of 0.15 (Figure 1). 

### 2.2. Mechanical Properties 

**Figure 2:**
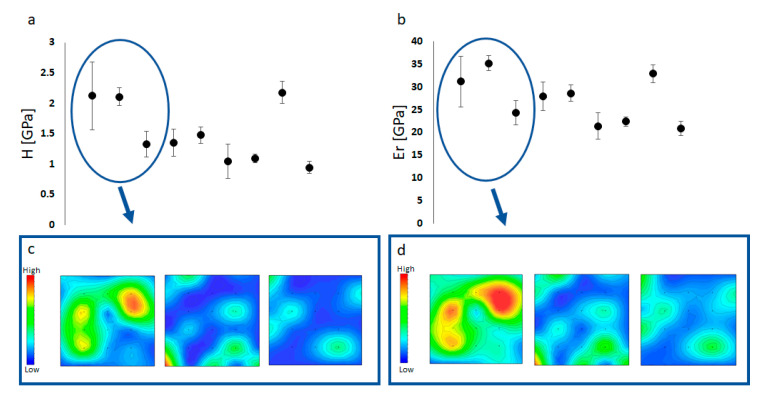
Mechanical properties of *Pneophyllum cetinaensis* measured by nanoindentation. (**a**) Hardness measurements of the nine specimens analysed (specimens in numerical order one–nine starting from the left to right along the x axes). (**b**) Elastic modulus measurements of the nine specimens analysed (specimens in numerical order one–nine starting from the left to right). (**c**) Hardness partial maps (15 × 15 indents) of specimens one to three (starting from the left) inside the blue oval in panel (**a**). (**d**) Elastic modulus partial maps (15 × 15 indents) of specimens one to three (starting from the left) inside the blue oval in panel (**b**). (**a**,**b**) Error bars = standard errors; X axes = specimens. (**c**,**d**) Colour legends are given in units of GPa where red is hardest (**c**) and stiffest (**d**). High variability within the same specimen (**c**,**d**) is also clearly visible in the maps, where peaks in hardness are shown in red.

The hardness (Figure 2a,c) showed significant variability (p_H_ = < 0.001; p_Er_ = < 0.001, respectively) among specimens with an overall average of 1.71 ± 1.58 GpA. Specimen 8 was significantly different from specimen 6 (Kruskal–Wallis; Dunn’s; Sp_8_vsSp_6_: *p* = 0.019), 9 (Kruskal–Wallis; Dunn’s; Sp_8_vsSp_9_: *p* = 0.0023) and 7 (Kruskal–Wallis; Dunn’s; Sp_8_vsSp_7_: *p* = 0.004), while specimen 2 was significantly different from specimen 7 (Kruskal–Wallis; Dunn’s; Sp_8_vsSp_7_: *p* = 0.039). 

The elastic modulus (Figure 2b,d) also showed a high variability among specimens, with specimen 7 significantly different from specimen 2 (Kruskal–Wallis; Dunn’s; Sp_2_vsSp_7_: *p* = <0.001) and specimen 8 (Kruskal–Wallis; Dunn’s; Sp_8_vsSp_7_: *p* = 0.030). The overall average of the elastic modulus was 29.7 ± 18.0 GPa. Like the hardness the elastic modulus was not homogeneous within specimens.

### 2.3. Elements Composition

**Figure 3:**
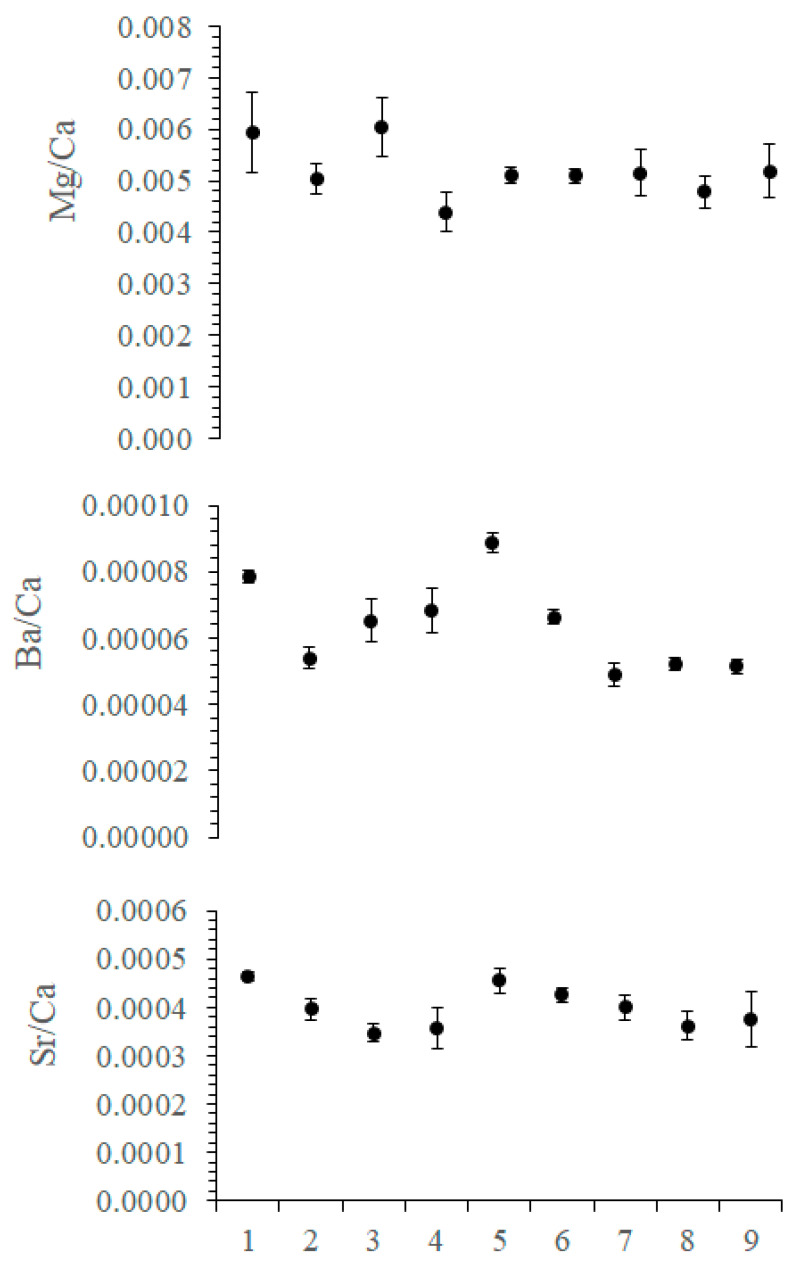
Element concentrations (µg/g) of nine specimens of *Pneophyllum cetinaensis*. X axes identify the specimens. The graph shows the average ± SD of all specimens.

The investigated elements are summarized in Table S2 and Figure 3. Elements were chosen on the bases of either importance for the structural integrity (Mg/Ca) or importance as a proxy (Sr/Ca for temperature; Ba/Ca for coastal sediment transport, freshwater discharge, salinity, and nutrients distributions) in marine coralline algae. There is a significant difference for some of the elements between different specimens.

Mg/Ca concentrations (µg/g) do vary significantly among specimens, with an overall average of 0.110 ± 0.31. However, there is a significant difference between specimens nr. seven, nr. four (Kruskal–Wallis; Dunn’s; Sp_4_vsSp_7_: *p* = 0.003), and nr. two (Kruskal–Wallis; Dunn’s; Sp_2_vsSp_7_: *p* = 0.039).

Ba/Ca concentration (µg/g) ratios have an overall average of 6.3 × 10^−5^ ± 1.7 × 10^−5^ (µg/g). Specimen nr. four was significantly different from specimens nr. six (Kruskal–Wallis; Dunn’s; Sp_4_vsSp_6_: *p* = 0.001), nr. eight (Kruskal–Wallis; Dunn’s; Sp_4_vsSp_8_: *p* = 0.004) 7 (Kruskal–Wallis; Dunn’s; Sp_4_vsSp_7_: *p* = 0.007), and nr. one (Kruskal–Wallis; Dunn’s; Sp_4_vsSp_1_: *p* = 0.001).

Sr/Ca concentration (µg/g) ratios were also very similar between specimens with an overall average of 3.95 × 10^−4^ ± 7.99 × 10^−5^. Only specimen nr. three was significantly different from specimen nr. one (Kruskal–Wallis; Dunn’s; Sp_3_vsSp_1_: *p* = 0.047) and specimen nr. 5 (Kruskal–Wallis; Dunn’s; Sp_3_vsSp_1_: *p* = 0.043).

**Figure 4:**
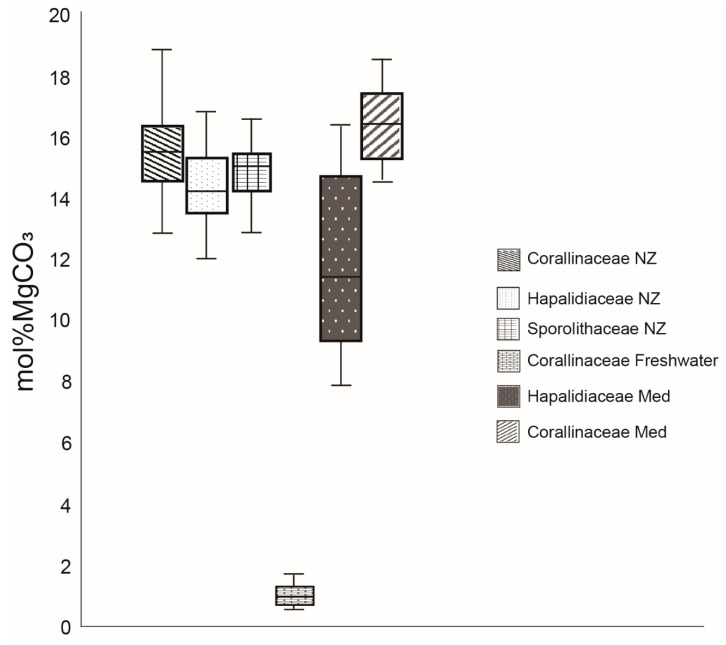
mol% MgCO_3_ of marine coralline algae from different families and regions (New Zealand (NZ) and Mediterranean Sea (Med)) and the freshwater algae *Pneophyllum cetinaensis.* Data of the seawater coralline algae were collected from: [17,37,38].

Magnesium concentrations in all the specimens of *Pneophyllum cetinaensis* were ~10 times lower than the concentrations found in coralline algae living in seawater (Figure 4). With the magnesium concentrations of *Pneophyllum cetinaensis,* the family of the Corallinaceae features the highest and the lowest concentrations of mol% MgCO_3_ (Figure 4) among all calcifying red algae living in temperate regions.

## 3. Discussion

All the measurements carried out during this study showed some significant variability among specimens. The thallus of *Pneophyllum cetinaensis* is layered and arranged in superimposed flattened branches (Figure 1) which grow at different times (i.e., months). We were unable to determine the specific time of growth for each layer and therefore, the variability of the elements and structural measurements between specimens most likely reflects the different physico–chemical parameters of the Cetina River during the growth of the different layers. 

There is a positive correlation between the magnesium content and hardness in biogenic calcite [19,39,40], however the magnesium concentrations in adult specimens of *Pneophyllum cetinaensis* were ~10 times lower (average of 0.97 molMg% ± 0.02 SD) than the concentrations found in coralline algae living in seawater (average of 14.7 molMg% ± 1.3 SD) [37,38]. Magnesium incorporation in coralline algae is determined by water temperature [24,41,42,43], phylogeny [17] and the water Mg/Ca ratio [13,18]. While temperature and phylogeny did not vary within our study, the Mg/Ca ratio of the ambient water in Cetina River was the driving factor of such a low magnesium content (Mg/Ca: 0.10 ± 0.66 SD mg/L). As conditions of elevated Mg/Ca ratios can gives highly variable calcites but low Mg/Ca ratio can only give low Mg Calcites [44]. The structural integrity of *Pneophyllum cetinaensis* showed a highly heterogeneous distribution of elastic modulus and hardness within and between the specimens. Surprisingly, Hardness (H) and Elastic modulus (Er) in *Pneophyllum cetinaensis* are within the same range of value measured in different coralline algae species living in different marine environments (intertidal: [45], subtidal: [46]). Cristallographic size and texture play an important role in the optimization of the calcite material properties [47]. However, in biominerals, small variation in geometrical parameters and crystal size are very common, therefore the composite behaviour of the biostructure does not usually depend on small variation in structural geometry [48]. Mechanical anisotropy due to the heterogeneous structure of coralline algae is thought to increase the risk of fractures but the high magnesium content improves the hardness of the thallus [49]. The process behind the hardening of the biogenic calcite by Mg^2+^ substitution is related to the creation of lattice distortion due to the smaller size of Mg^2+^ compared with calcium [40], which will hinder dislocation motion and increase hardness. However, there are other components apart from Mg^2+^ that help increase the hardness in biogenic calcite [50]. Via model dynamics, Cote’ t al. [50] proved that biogenic impurities, such as amino acids, decrease the strain required to induce plastic deformation in calcite, consequently increasing hardness and perforation resistance. Therefore, the mismatch of H, Er and magnesium concentration could be linked to other added impurities that might contribute to the enhanced hardness of biogenic calcite.

*Pneophyllum cetinaensis* is the only known living low-Mg calcite coralline algae. Despite the riverine environment, the low Mg/Ca ratio in *Pneophyllum cetinaensis* is very close to the ratio predicted from seawater (Ries 2006) calibrations considering the measured Mg/Ca ratio in the Cetina River. The possibility of the existence of low-Mg calcite coralline algae was already investigated in some laboratory experiments looking at late Cretaceous seas [51]. After investigating the changes in the Mg/Ca ratio of *Amphiroa* genus growing in Mg/Ca=1 artificial seawater, [52] concluded that many taxa that now produce high-Mg calcite, produced low-Mg calcite in late Cretaceous seas. In *Pneophyllum cetinaensis*, the capability to maintain the low-Mg calcite polymorph that is less susceptible to dissolution at lower pH [53], has probably evolved to cope with the pH fluctuations occurring in the Cetina river. The pH of the study site is on average 8.19 ± 0.21 with occasional decrease in pH as low as 7.0 during summer. A study on *Lithothamnion glaciale* [19] showed a significantly lower magnesium concentration in specimens growing at pH 7.9. 

Both strontium and barium element concentrations in *Pneophyllum cetinaensis* are roughly two orders of magnitude lower than the average Sr/Ca and Ba/Ca ratios in marine coralline algae ([54] Sr/Ca: from 6.0 × 10^−3^to 9.0 × 10^−3^; Ba/Ca: from 2.0 × 10^−5^ to 0.4 × 10^−5^).

The low Sr^2+^ values are expected since Mg^2+^ should facilitate the uptake of Sr^2+.^ In fact, the incorporation of smaller Mg^2+^ in the calcite lattice may distort the crystal lattice subsequently facilitating the uptake of larger ions such as Sr^2+^ [19]. Therefore, the low Sr^2+^ values are likely caused by the low magnesium concentrations in *Pneophyllum cetinaensis*.

Concentrations of barium are generally higher in rivers than in seawater (50 μg/L vs. 6 μg/L Ba^2+^; [55]) as a result of chemical weathering in their catchments, and for this reason, one might have expected a higher Ba/Ca ratio in *Pneophyllum cetinaensis* compared to marine coralline algae. However, the mechanisms of barium enrichment in coralline algae are still not fully understood. Several studies on marine coralline algae report either positive, negative or no correlation between Ba/Ca and freshwater input (for examples nutrients [31]). For instance, Hetzinger et al. [56] showed a positive correlation between sea surface salinity and Ba/Ca, while a study carried out by Chan et al. [35] concluded that there is a negative correlation between Ba/Ca and salinity. Moreover Caragnano et al. [31] showed no correlation between precipitation and Ba/Ca ratio. Another possible explanation of our finding can be related to the higher sulphate ion activity in seawater compared to freshwater [57]. Since no analyses were carried out on the barium concentration in the Cetina River waters, we cannot exclude that the low Ba/Ca ratio could be related to an unusually low barium concentration in the Cetina River. 

## 4. Materials and Methods 

### 4.1. Sample Collection

Specimens of *Pneophyllum cetinaensis* were collected in the Cetina River (southern Croatia) at a depth of 0.5 m in December 2013 at Otok Ljubavi (43°26.180′ N–16°45.785′ E). The Cetina River is a typical permanent karst river discharging into the Adriatic Sea. *Pneophyllum cetinaensis* is present throughout almost the entire length of the Cetina River from 0 to 300 m above sea level, reaching about 75 km from the river mouth. The Cetina River physico–chemical characteristics at the site of collection were: pH = 8.21 ± 0.02 SD (NBS scale), salinity < 0.5, temperature: 10.20 ± 0.08 SD °C (annual average; 12.9 ± 3.3 SD °C), Mg = 7.52 ± 4.11 SD, mg/L, Ca = 68.86 ± 6.18 SD mg/L.

**Figure 5:**
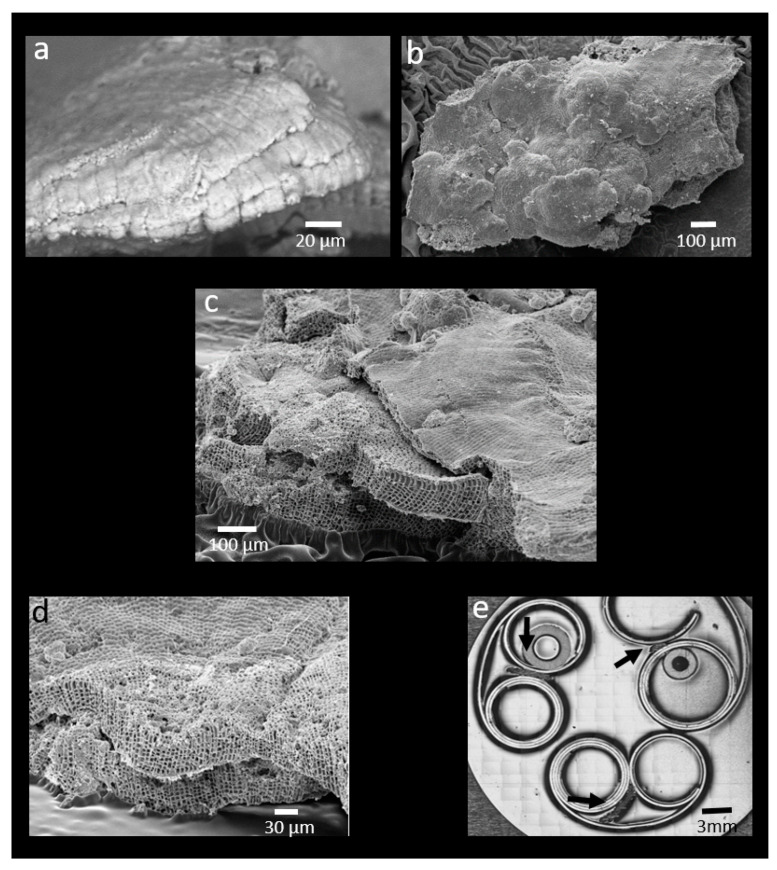
Structure and sample preparation of *Pneophyllum cetinaensis* (**a**–**c**). *Pneophyllum cetinaensis* layered thallus, arranged in superimposed flattened or curved fragile branches. (**d**) Example of thallus layer used for the analysis. (**e**) Stub with polished *Pneophyllum cetinaensis* thalli (black arrows), ready to be measured.

Prior to the mechanical properties and trace elements analysis carried out at the University of Portsmouth, nine specimens were embedded in epoxy resin (EpoFix Kit, batch no: 8134-01, Struers ApS, Ballerup, Denmark) and gently polished (Micropolish Alumina, Buehler, Esslingen, Germany). Analyses were carried out on the internal layers of the algae (Figure 5d). The LA-ICPMS spots size and the Nano indentation grid allowed us to analyse the entire layers.

### 4.2. SEM Analysis

Two different SEM were used to create Figure 1. SEM images of Figure 5a,b,d were taken at the University of Portsmouth with an SEM suitable to analyse uncoated samples (EVO MA10 with a W filament electron source, Zeiss, Oberkochen, Germany). This SEM was used to allow the transfer of samples between the LA-ICP-MS and SEM preventing interferences from sample coating. Prior to cleaning with isopropanol, each mount was fixed on a stab using double coated carbon conductive tabs. Samples were placed into the SEM and a variable pressure (VP) vacuum of 28 Pa was applied.

Images were taken at 20 kV electron high tension (EHT), and a working distance (WD) of ~7 mm, using a probe current of 200 pA, a scan speed of 20.5 s, a magnification of 1–2 K and a line average noise reduction in the Backscatter (HDBSD) mode.

SEM images of Figure 5c,d were taken at the University of Trieste. Fragments were mounted on aluminium stubs and coated with gold/palladium (with S150 Sputter Coater, Edwards, Crawley, UK) prior to viewing with a LEICA Steroscan 430i (Cambridge, UK) at 20 kV.

### 4.3. XRD/Phase Identification

For XRD evaluation, the sample was slightly crushed with an agate mortar and prepared into a special single-crystal silicon cavity sample holder via front loading method. Due to the limited amount of sample, only one preparation was possible. 

The XRD measurement was performed at a D8 Advance with DaVinci design diffractometer (Bruker AXS, Karlsruhe, Germany) with the following parameters: angle range 10–70° 2θ; step size 0.0112° 2 θ; integration time 0.3 s; divergence slit 0.3°; radiation: Cu K_α_; generator settings: 40 mA, 40 kV. Rietveld refinement was conducted with software TOPAS V5 (Bruker AXS, Karlsruhe, Germany). For the refinement of calcite, the structure ICSD #80869 [58] was applied together with a Chebychev polynomial of 3rd order for the background. Refined parameters were scale factor, lattice parameters, cry size (Lorentz contribution) and microstrain (Gauss contribution). 

### 4.4. Mechanical Properties

Nanoindentation was performed at the University of Portsmouth using a depth sensing indentation instrument (Nano Test Platform 3, Micro Materials Ltd., Wrexham, UK). This pendulum-based nanoindentation system is extensively explained elsewhere [59,60]. Indentations were performed using a Berkovich diamond indenter in load-controlled mode. Maximum loading force was set to 5 mN, loading and unloading rates were kept constant, with loading and unloading rates each set to 0.01 mN s^−1^, and a dwell time of 30 s was selected at maximum load to reduce the influence of creep. A matrix of 60 to 120 indents with a 50 μm space between each indent was imprinted onto the algal surface to map the distribution of mechanical properties within each specimen. The defined number of indents allowed us to measure properties over the whole or half of the algal sample, depending on its size. Before and after indentation, all samples were imaged using an integrated optical microscope to identify the position of an indent. During nanoindentation experiments, a series of force vs. displacement curves were recorded. The analysis was performed using analytical software provided by MicroMaterials, where the unloading portion of the curve was fitted to a power law function [61] to determine the hardness and elastic modulus of algae samples. 

The physical aspects of nanoindentation analysis are explained in detail by Beake [59,60], and therefore will not be repeated here. Sample hardness (*H*) was calculated from the maximum load (*F*_max_) and the projected area of contact (*A*_c_), determined through a series of indentations at different loads on a calibration sample of fused silica, using the equation:

(1) $$H=\frac{F_{\max}}{A_{c}}$$

Young’s modulus (or elastic modulus), *E*, of the sample can be determined using the equation:

(2) $$\frac{1}{E_{r}}=\frac{1-v^{2}}{E}+\frac{1-v_{i}^{2}}{E_{i}}$$

where *ν* is the Poisson’s ratio of the sample, *E_r_* is the reduced modulus of the sample derived from the load vs. displacement curves [61], ν*_i_* is the Poisson’s ratio of the indenter (0.07) and *E_i_* is the Young’s modulus for the indenter (1141 GPa). As Poisson’s ratios of the algae are not known, the reduced indentation modulus (*E_r_*) will be reported in this paper instead.

Maps of elastic modulus and hardness were generated to determine the distribution of the mechanical properties. These maps were further processed by eliminating values obtained on epoxy resin as well as where surface defects interfered with points of measurement. For statistical analysis, histograms of modulus and hardness were also obtained and values for each indent were averaged across the individual specimens for each treatment. 

### 4.5. Element Analysis

#### Instrumentation, Operating Conditions and Data Reduction

Trace element analyses were conducted in two sessions at the University of Portsmouth. The first analytical session was carried out using an Agilent 7500cs Quadrupole ICP-MS coupled with a Nd:YAG 213 nm New Wave solid-state laser ablation system. The second session utilized a RESOlution 193 nm ArF excimer Laser with a Laurin Technic S155 Ablation cell (Australian Scientific Instruments, Canberra, Australia) coupled to an Analytic Jena Plasma Quant MS Elite ICP-MS. Background and signal counts were integrated, time-drift corrected and reduced to concentrations using the SILLS [62] and Iolite 3.4 software packages [63], respectively, for the first and second sessions (Table S1). Synthetic silicate glass reference materials NIST SRM 610 as well as NIST SRM 612 were used for instrumental calibration and as primary and secondary standards (Table S2). Synthetic calcium carbonate USGS MACS-3 was also used as secondary standard and analysed in the same conditions as the unknowns. Detection limits (99% confidence) of the NIST glasses for spot measurements were: Mg = ~3 ppm for session 1 and ~28 ppm for session 2, Ca = ~30 ppm for session 1 and ~100 ppm for session 2. The internal standard element used for normalization of the data was ^43^Ca. All reference materials were ablated prior, in the middle, and after sample ablation. Following every 6th sample analysis, one analysis of NIST SRM 610 was added to correct for time-dependent drift of mass discrimination and instrument sensitivity.

Final elemental composition ratios in this study were calculated as a “mean count rate” including standard deviation of five drift and background corrected single ablation spot analysis for each of the nine replicates. This method is commonly used for LA-ICP-MS data reduction [64]. Magnesium contents obtained throughout the course of this study are reproducible within 10%, of the GeoReM database recommended values (reproducibility: Table S1, accuracy: Figures S1 and S2).

### 4.6. Statistical Analysis

All analyses were run in SPSS statistic 24 (IBM Corp., Armonk, NY, USA, 2016) and data sets were tested for normality and homogeneity prior to further analysis. All data sets lacking normal distribution (elastic modulus, Hardness, Mg/Ca and Sr/Ca) were analysed using a non-parametric test (Kruskal–Wallis H). The Ba/Ca data sets were analysed using multiple single comparisons of one-way analysis of variance (ANOVA).

## 5. Conclusions

With the low-Mg calcite (~10 times lower than seawater coralline algae) thallus of *Pneophyllum cetinaensis* the family of the Corallinaceae has the highest and the lowest concentration of Mg among calcifying red algae living in temperate regions. The low-Mg calcite of the thallus is likely to be an adaptation to Cetina River carbonate chemistry. The adaptation of *Pneophyllum cetinaensis* to live in a low Mg/Ca environment reveals the likely capability of coralline algae to maintain the low-Mg calcite polymorph (less susceptible to dissolution at lower pH) as a strategy to cope with seawater acidification due to climate change. Although there is a positive correlation between Mg and hardness in biogenic calcite, hardness and elastic modulus in *Pneophyllum cetinaensis* are within the same range of values measured in different marine coralline algae species which could be explained by the presence of biogenic impurities. The Sr/Ca and Ba/Ca ratios in *Pneophyllum cetinaensis* are roughly two orders of magnitude lower than average Sr/Ca and Ba/Ca ratios in marine coralline algae. The explanation for the lower Ba/Ca of *Pneophyllum cetinaensis* should be investigated, focusing on the analysis of Ba concentration in the Cetina River and on the mechanism of Ba incorporation in the low-Mg calcite skeleton.

## Supplementary Materials

The following are available online at https://www.mdpi.com/2223-7747/9/9/1089/s1, Table S1: Laser and ICP-MS operation parameters, Table S2: Reproducibility of reference materials USGS MACS-3 and NIST SRM 612 for each element measured and analyzed in this study. Figure S1: Accuracy of the reference material USGS MACS-3, Figure S2: Accuracy of the reference material NIST SRM 612. Table S3: Element ratios of the nine specimens of *Pneophyllum cetinaensis*.
