# Supplementary material for: Structural and Elemental Analysis of the Freshwater, Low-Mg Calcite Coralline Alga Pneophyllum cetinaensis
Source: Plants (Basel). 2020 Aug 24;9(9):1089. doi: 10.3390/plants9091089 (PMC7570384; doi:10.3390/plants9091089)
Supplement: Supplementary file 1 [file plants-09-01089-s001.zip › plants-891785-supplementary-xml.docx]

**Table S1.** Laser and ICP-MS operation parameters.

| **Laser parameters** | | |  |
| --- | --- | --- | --- |
|  | **Session 1** | **Session 2** |  |
| Wavelength | 213 nm | 193 nm |  |
| Fluence | 3.5 – 4.0 J cm^−2^ | 3.0 J cm^−2^ |  |
| Laser Energy | 55% | 14.0 mJ |  |
| Carrier gas | Helium (He) | Helium (He) + Argon (Ar) |  |
| Ablation style | Static | Static |  |
| Ablation spot size | 55 μm for glasses and 80 μm for unknowns | 55μm |  |
| Ablation sequence | 75 sec (background, laser warming, acquisition, washout) | 65 sec (background, acquisition, washout) |  |
| Repetition rate | 10 Hz | 5 Hz |  |
| **ICP-MS parameters** | | | |
|  | Session 1 Session 2 | |  |
| RF power | 1450 W | 1300 W |  |
| Plasma gas | 16 L min^−1^ | 10 L min^−1^ |  |
| Auxiliary gas | 1.0 L min^−1^ | 1.65 L min^−1^ |  |
| Nebulizer flow | 0 L min^−1^ | 0.87 L min^−1^ |  |
| Sampling depth | ~5.0 mm | ~8.5 mm |  |
| Detector | Single collector | Single collector |  |
| Dwell time per mass | 10 ms | 10 ms |  |

**Table S2.** Reproducibility of reference materials.

|  | **MACS-3** | **NIST 612** |
| --- | --- | --- |
| Mg [ppm] | 2089.61 ± 451.03 | 64.42 ± 6.37 |
| Ca [ppm] | 405926.27 ± 31595.49 | 82995.1 ± 4273.56 |
| V [ppm] | 44.37 ± 7.86 | 43.87 ± 2.77 |
| Cr [ppm] | 115.92 ± 19.41 | 41.41 ± 2.38 |
| Fe [ppm] | 10972 ± 2063.16 | 77.94 ± 43.08 |
| Zn [ppm] | 105.04 ± 13.61 | 42.38 ± 4.34 |
| Sr [ppm] | 7778.75 ± 661.97 | 78.83 ± 5.66 |
| Cd [ppm] | 43.22 ± 7.82 | 31.36 ± 3.82 |
| Ba [ppm] | 64.69 ± 6.42 | 40.21 ± 7.16 |

USGS MACS-3 and NIST SRM 612 (Total Mean ± 2SD) for each element measured and analysed in this study.


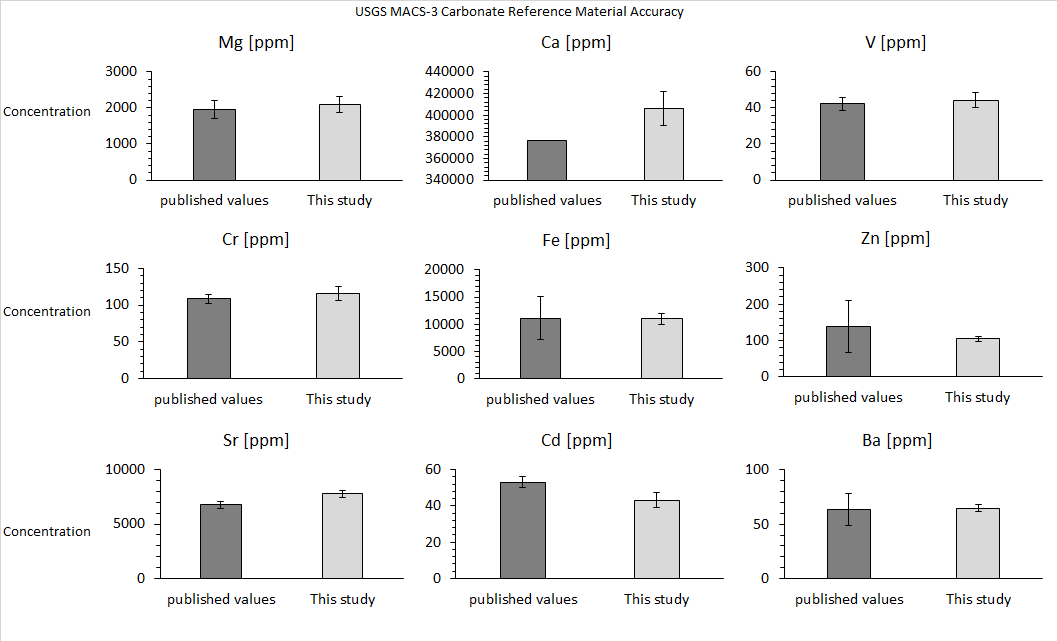


**Figure S1.** Accuracy of the reference material USGS MACS-3 for each measured and analysed trace element. Dark grey bars represent AV ± SD [ppm] of published data retrieved from the GeoReM database. Light grey bars represent AV ± SD [ppm] of trace elements in this study.


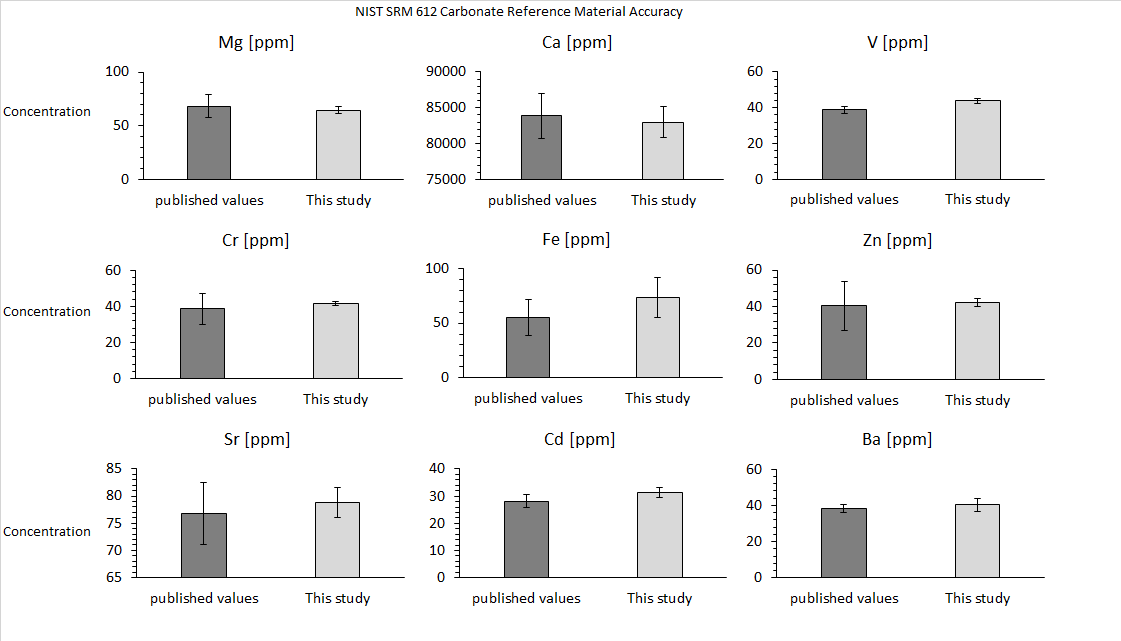


**Figure S2.** Accuracy of the reference material NIST SRM 612 for each measured and analysed trace element. Dark grey bars represent AV ± SD [ppm] of published data retrieved from the GeoReM database. Light grey bars represent AV ± SD [ppm] of trace elements in this study.

**Table S3.** Element ratios of the nine specimens of *Pneophyllum cetinaensis.*

| ***Element ratio (*µ*g/g)*** | | | | | | | | | | | | |
| --- | --- | --- | --- | --- | --- | --- | --- | --- | --- | --- | --- | --- |
|  | ***Sample*** | **Cd/Ca** | | **Cr/Ca** | | **V/Ca** | | **Zn/Ca** | | **Fe/Ca** | |  |
|  |  | Average | SE | Average | SE | Average | SE | Average | SE | Average | SE |  |
|  | 1 | 0.00000025 | 0.0000000294 | 0.0000247 | 0.00000693 | 0.00000301 | 0.000000523 | 0.000101 | 0.0000184 | 0.0000821 | 0.0000205 |  |
|  | 2 | 0.000000254 | 0.0000000511 | 0.0000369 | 0.00000921 | 0.00000133 | 0.000000855 | 0.0000154 | 0.00000248 | 0.000545 | 0.000323 |  |
|  | 3 | 0.000000452 | 0.0000000754 | 0.0000298 | 0.00000513 | 0.00000254 | 0.000000618 | 0.0000731 | 0.0000256 | 0.000485 | 0.000207 |  |
|  | 4 | 0.00000033 | 0.0000000279 | 0.0000222 | 0.0000017 | 0.000000479 | 0.000000124 | 0.00000629 | 0.00000186 | 0.000268 | 0.0000228 |  |
|  | 5 | 0.000000359 | 0.0000000337 | 0.0000204 | 0.0000037 | 0.000000509 | 0.000000144 | 0.0000661 | 0.0000289 | 0.000251 | 0.0000235 |  |
|  | 6 | 0.000000337 | 0.0000000254 | 0.0000178 | 0.000000449 | 0.000000348 | 0.000000114 | 0.0000166 | 0.00000309 | 0.000103 | 0.00000841 |  |
|  | 7 | - | - | - | - | - | - | 0.0000308 | 0.00000515 | 0.000177 | 0.000177 |  |
|  | 8 | - | - | - | - | - | - | 0.0000269 | 0.00000658 | 0.000872 | 0.000125 |  |
|  | 9 | - | - | - | - | - | - | 0.000028 | 0.0000107 | 0.000267 | 0.000163 |  |
